# Retraction: Frondoside A Suppressive Effects on Lung Cancer Survival, Tumor Growth, Angiogenesis, Invasion, and Metastasis

**DOI:** 10.1371/journal.pone.0291478

**Published:** 2023-09-08

**Authors:** The PLOS ONE Editors

After this article [1] was published, concerns were raised about the mouse tumor sizes reported in Fig 3, as well as the experimental endpoint. Specifically:

- The charts in Fig 3A and B of the article [1] appear to report tumor sizes of up to 5500 mm^3^, with a standard error of approximately 1000 mm^3^.
- The charts in Fig 3C-Fappear to show tumor burdens greater than 10% of body weight.

The authors provided underlying individual-level tumor volume and mouse weight data in post-publication discussions. The data showed that for the Fig 3 experiment, individual mouse tumor sizes were up to 8980.2 mm^3^ and many individual mouse tumor sizes were over 2000 mm^3^.

The corresponding author stated that the objective in the experiment presented in Fig 3 was to assess whether the tested compound could effectively reduce the growth of highly tumorigenic and fast-growing lung tumors, providing a closer approximation to the severity of this cancer in humans. They noted that humane endpoints were in place during the experiment; animals were monitored daily by a veterinarian; the study strictly adhered to fundamental principles outlined in the applicable guidelines; and the authors took measures to minimize any potential distress or pain experienced by the animals. According to the authors, none of the humane endpoint criteria were observed, no signs of pain were observed, and no painkillers (analgesics) were administered. However, due to a concern about weight loss in one mouse in the control group, the experiment was terminated before the planned experimental endpoint.

PLOS consulted an expert in animal research ethics and welfare about this case. The expert confirmed that the tumor sizes reported for this study exceed size standards in the US and internationally. They also raised concerns about aspects of the humane endpoint criteria, and about the lack of specific data on health monitoring outcomes.

Similar to the current policy, the journal’s Animal Research policy in place in 2013 required that studies involving animals must have been conducted according to internationally accepted standards. Based on our assessment and the input received from the consulted animal welfare expert, *PLOS ONE* concluded that the study did not meet the journal’s animal research ethics standards. Therefore, the *PLOS ONE* Editors retract this article.

The editors regret that these concerns were not addressed prior to the article’s publication.

SA did not agree with the retraction. KA, AG, MAAS, MB, PC, TT, TEA, and ODW either did not respond directly or could not be reached.
